# Why don't all birds lay early? Causes and consequences of breeding timing for a long‐lived migratory seabird

**DOI:** 10.1111/1365-2656.70363

**Published:** 2026-10-10

**Authors:** Katrina Siddiqi‐Davies, Natasha Gillies, Hector Bailey, Sarah Bond, Annette Fayet, Holly Kirk, Lewis Lancaster‐Reeves, Joe Morford, Oliver Padget, Akiko Shoji, Martyna Syposz, Joe Wynn, Tim Guilford

**Affiliations:** ^1^ Department of Biology University of Oxford Oxford UK; ^2^ Department of Animal and Plant Sciences University of Sheffield Sheffield UK; ^3^ Norwegian Institute for Nature Research Trondheim Norway; ^4^ School of Molecular and Life Sciences Curtin University Perth Western Australia Australia; ^5^ University of Lausanne Lausanne Switzerland; ^6^ School of Environmental Sciences University of Liverpool Liverpool UK; ^7^ Graduate School of Environmental Studies Nagoya University Nagoya Japan; ^8^ Department of Vertebrate Ecology and Zoology University of Gdańsk Gdańsk Poland

**Keywords:** breeding phenology, carry over effects, chick provisioning, climate change, lay date, migration, migratory timing, seabird

## Abstract

1. The relationship between earlier laying and higher breeding success is well‐established in avian species. However, a wide distribution of breeding timings is often maintained within a population, and the causes and consequences of this variation require further exploration, particularly as many avian species advance breeding phenology in response to climate change.
2. Using a 16‐year dataset combining colony observations and biologging of Manx shearwaters (*Puffinus puffinus*), we show that earlier laying does not always result in higher breeding success, with later laying becoming less costly in recent years.
3. We additionally report a slight advancement in breeding timing within breeding pairs, despite finding no temporal trends in spring bloom onset. Nonetheless, birds adjusted lay date in response to interannual variation in phytoplankton bloom timing, and years with stronger Celtic Sea blooms exhibited stronger selection for early breeding.
4. Breeding timing was also influenced by repeatable individual differences in migratory behaviour, with birds undertaking generally earlier, shorter migrations to less southerly destinations laying earlier across years.
5. However, lay date was not determined solely by individual behaviour. In this long‐lived species with stable pair bonds, the arrival date of the second‐returning bird constrained advancement in laying.
6. This study suggests that variation in breeding timing is maintained through the combined effects of fluctuating selection, persistent individual differences, variation in non‐breeding duration and pair‐level processes.

## Graphical abstract

Why don't all birds lay early? Our 16‐year study of Manx shearwater behaviour shows that selection on breeding timing fluctuates among years. We also find that lay dates are influenced by persistent individual differences, variation in non‐breeding duration, and pair synchrony, potentially constraining breeding phenological advancement under ongoing climate change.

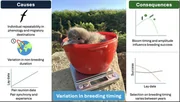

## Crynodeb

1. Mae'r berthynas rhwng dodwy cynharach ac uwch lwyddiantbridio wedi'i hen sefydlu mewn rhywogaethau adar. Fodd bynnag, yn aml o fewn poblogaeth, cynhelir dosbarthiadeang o amseriadau bridio ac mae angen ymchwil bellach iachosion a chanlyniadau'r amrywiad hwn, yn enwedig ganfod llawer o rywogaethau adar yn symud eu ffenoleg bridioymlaen mewn ymateb i newid hinsawdd.
2. Gan ddefnyddio set data 16 mlynedd, sydd yn gyfuniad o arsylwadau cytrefol â bio‐gofnodi Aderyn‐Drycin Manaw (*Puffinus puffinus*), dangoswn na fydd dodwy cynharach bob amser yn gwella llwyddiant bridio gyda dodwy hwyrach ynprofi'n llai costus yn ystod y blynyddoedd diweddar.
3. Adroddwn yn ychwanegol symudiad bach ymlaen ynamseriad bridio parau bridio er na chanfuom unrhywdueddiadau tymhorol yn nechreuad blodeuo'r gwanwyn. Serch hynny, bydd adar yn newid eu dyddiad dodwy o flwyddyn i flwyddyn mewn ymateb i amrywiad yn amseriadblodeuo'r ffytoplancton ac fe arddangosodd blynyddoedd o flodeuo cryfach yn y Môr Celtaidd ddetholiad cryfach ifridio'n gynharach.
4. Hefyd roedd gwahaniaethau ailadroddadwy mewn ymddygiadmudol yn dylanwadu ar amseriad bridio. Roedd adar a fudentyn gynharach, i leoliadau llai deheuol, yn dodwy'n gynharachdros gyfnod o flynyddoedd.
5. Fodd bynnag ni phenderfynid dyddiad dodwy gan ymddygiadunigolion yn unig. Yn y rhywogaeth hirhoedlog hon, gydarhwymau pâr sefydlog, cyfyngai dyddiad dychwelyd ail aderyn y pâr ar y gallu i ddodwy'n gynharach.
6. Mae'r astudiaeth hon yn awgrymu bod amrywiad mewnamseru bridio yn cael ei gynnal drwy effeithiau cyfunedig:detholiad anwadal, gwahaniaethau unigol parhaol, amrwyiadyn hyd y cyfnod heb fridio a phrosesau lefel‐pâr.

## 1 INTRODUCTION

The timing of breeding is a well‐known determinant of overall breeding outcomes in birds, with earlier laying generally correlated with high reproductive success (Bennett et al., 2022; Ollason & Dunnet, 1978; Perrins, 1970; Vergara et al., 2007). Several mechanisms have been proposed to explain this pattern, which broadly describe interactions between an individual's phenotypic and environmental quality (Sutton & Freeman, 2023; Verhulst & Nilsson, 2008). For example, earlier laying can be a correlate of higher fitness, and so higher reproductive success may be driven by factors such as an individual's condition (Burant et al., 2022; Descamps et al., 2011; Winkler et al., 2020), competitive ability (Kokko, 1999), age and experience (Coulson, 1966; Culina et al., 2020; Sánchez‐Macouzet et al., 2014). However, the timing of laying itself can have a role in reproductive outcomes. For example, earlier laying may allow better alignment with seasonal peaks in productivity for chick or self‐provisioning (Both et al., 2010; Lameris et al., 2018; Yarys et al., 2021), facilitate individuals to secure quality nest sites (Kokko, 1999; Samplonius & Both, 2017; Smith et al., 2018), and in cases where species lay multiple clutches, allow time for further breeding attempts if the first fails (Morrison et al., 2019).

Given the physical costs associated with reproduction and the selective pressure to produce high‐quality offspring, if laying earlier than other birds is mostly beneficial, then why do many individuals within a population lay later (Perrins, 1970)? This could be explained as a gradient in the phenotypic quality of individuals from the earliest to the latest‐laying bird (Burr et al., 2016). However, alongside the known benefits, there may be occasional costs to earlier laying which prevents this from being the dominant strategy (Sutton & Freeman, 2023). For example, individuals breeding early may have insufficient time to replenish energy reserves following migration (Daan et al., 1986), or the earliest birds may be more vulnerable to extreme early season variations in weather that may hamper the breeding attempt or cause adult mortality (Shipley et al., 2020). As selection is expected to act on total lifetime fitness rather than annual breeding success alone (Daan et al., 1990; Drent & Daan, 1980), the balance of these costs and benefits is likely to depend on both environmental conditions and individual quality. Individuals in higher nutritional condition may be better able to withstand the costs associated with early reproduction (Hadfield & Reed, 2022; Pettifor et al., 1988).

Alternatively, interannual or longer phased changes in climate may shift the optimal lay date earlier or later between years (Bertacchi et al., 2016). Therefore, a heterogenous environment may favour earlier breeders in some breeding seasons and later breeders in others, maintaining greater among‐individual variation in lay date than would be expected under a stationary optimum (Bailey et al., 2022; Capilla‐Lasheras et al., 2022). While long‐term avian studies support the idea that shifting resource phenology may alter optimal breeding timing (Hipfner, 2008; Visser et al., 2012; Zhemchuzhnikov et al., 2021), we do not fully understand how this translates into selection on breeding time between years (Visser & Gienapp, 2019).

In recent years, multiple species of birds have seen a shift towards earlier lay dates, which is hypothesised to be a response to match advancing resource phenology with climate change (Andreasson et al., 2023; Both et al., 2004; Charmantier et al., 2008; Shipley et al., 2020). The mechanisms by which an individual defines their lay date between years may occur through behavioural or neuroendocrine processes, or some combination (Schaper et al., 2012). While some shifts in lay date might occur by population turnover, studies suggest that individuals have some plasticity in their lay date between years (Bonamour et al., 2019; Dobson et al., 2017; Nussey et al., 2005; Reed et al., 2006). Equally, plasticity is also evident in cases where lay date advances naturally within pairs due to experience or age (Brooke, 1990; Coulson, 1966). Identifying the factors that determine whether individuals lay earlier or later between years is fundamental for understanding their capacity to respond to climate change.

It may be difficult for long‐distance migrants to predict advances in resource phenology at the breeding site when they are exposed to a completely different set of environmental conditions overwinter (Gutiérrez‐Carrillo et al., 2024). Persistent individual differences in breeding and migratory phenology may occur, whereby some individuals are consistently earlier than others. However, carry over effects may also influence breeding timing, where environmental conditions during the non‐breeding period may cause individuals to breed earlier or later than would be expected based on their typical lay date (Fayet et al., 2016; Salton et al., 2015). Consequently, variation in migratory timing, distance and behaviour may contribute to differences in breeding timing among individuals (Bell et al., 2024).

Furthermore, in cases where reproductive strategy involves long duration partnerships, where both members of a pair reunite for breeding each year, lay date cannot be considered solely as an individual trait. Both members of a breeding pair must change their behaviour to commence breeding earlier. Pair synchrony may therefore limit the ability of breeding birds to advance their lay date with climate change (Gienapp et al., 2013). In a scenario where one bird may return to lay earlier and the other does not, an individual can either wait for its partner or form a new breeding pair. To reap the associated benefits of a long duration partnership, where divorce rates are typically low, and pair experience is associated with higher breeding success, re‐partnering is unlikely (Culina et al., 2015; Sánchez‐Macouzet et al., 2014). Additionally, re‐partnering requires finding a new partner, which may further delay breeding. From a global analysis of reproductive timing, seabirds are a group which appear to show little phenological response to shifting global temperatures and may be at increased risk of resource mismatch (Keogan et al., 2018). Although many seabirds maintain long duration partnerships (Visalli et al., 2023), there is little research that considers whether differences in partner arrival times may be a factor that constrains changes in phenology within breeding pairs.

In this study, we use a 16‐year geolocator dataset in combination with nest observations to investigate causes and consequences of lay date variation between breeding pairs, exploring interannual variability in the relationship between breeding timing and success and, secondarily, how migratory timing, destination, pair synchrony and experience underpin the lay date of a long‐distance seabird migrant. The Manx shearwater (*Puffinus puffinus*) is a burrow‐nesting procellariform seabird that forms long‐duration breeding pairings and typically lays a single egg each year (Brooke, 1990). The distance, duration and timing of their trans‐hemispheric migration are likely to constrain the timing of breeding (Freeman et al., 2013), especially as their partner must also return to commence breeding. As a relationship between earlier laying and reproductive success has previously been described for this species based on 11 years of data from the 1960s (Perrins, 2014), here we provide an updated investigation on the effect of lay date on metrics of reproductive success. We explore whether the timing of optimal egg laying may vary between years through an analysis of remotely sensed phytoplankton bloom metrics and finally report whether any longer phased changes in lay date have been observed over the 16 years of our study.

## 2 MATERIALS AND METHODS

### 2.1 Ethical approval

The Animal Welfare and Ethical Review Board at the University of Oxford (AWERB reference: APA/1/5/ZOO/NASPA/Guilford/ExodusForaging) ethically approved the deployment of geolocators on Manx Shearwaters. All data were collected in accordance British Trust for Ornithology (BTO) licences, including Special Methods approval for geolocator deployments and a C permit for ringing Manx shearwaters (permit number: C/6922). Access and research permission were granted by the Islands Conservation Advisory Committee (ICAC) and the wardens of Skomer Island.

### 2.2 Fieldwork

Fieldwork took place from 2009 to 2025 on Skomer Island, Wales, UK (51.7358° N, 5.2964° W), as part of a long‐term study of shearwater behaviour and navigation. Egg lay date was determined from daily nest inspections from April to July each year. To identify lay date variation within breeding pairs, the ID determined from British Trust for Ornithology (BTO) metal leg rings was recorded from each individual. Following hatching, from 2012 to 2025, a subsample of chicks (mean per year *n* = 64.10 ± 28.80 SD) were weighed daily to obtain measurements of peak mass, a key determinant of breeding success for this species, which is also indicative of adult chick provisioning intensity (Perrins, 2014). Burrows were monitored until the fledgling period in mid‐September to obtain information on which breeding pairs successfully reared a chick, and which abandoned or failed.

To measure migratory behaviour and pair synchrony, we used geolocators on breeding adults. During the study period, 418 geolocator/immersion loggers were deployed and retrieved, 194 of which had lay date manually recorded via nest inspection. Geolocators were attached to a custom‐built darvic leg ring using cable ties. As the study took place over a period of technology advancement, multiple geolocator models were used: BAS Mk 6, 9, 15, 19 (2.5 g), BAS Mk 13, 14, 18 (1.5 g) and MigrateTech intigeo C330, C250 (3.3 g), C65, C65‐Super (1 g) combined immersion and light loggers.

### 2.3 Chlorophyll phenology and variability metrics

We used the North Atlantic Ocean Colour Plankton, Reflectance, Transparency and Optics dataset (Bonelli et al., 2021; E.U. Copernicus Marine Service Information (CMEMS), 2025) to download data for the Celtic (10° W to 4° W and 48° N to 52.5° N) and Irish seas (8.5° W to −4° W and 52° N to 55.4° N). These represent the core breeding season foraging areas for Manx shearwaters from Skomer (Dean et al., 2013; Shoji et al., 2015) and were analysed separately as different regions may present separate phytoplankton phenology (Racault et al., 2012). For each region‐year, we derived a smoothed daily chlorophyll‐a curve from the satellite time series (Feb 1–Aug 31 window) using LOESS smoothing (span = 0.25) on the region‐mean daily values. From the smoothed curve, we calculated two primary metrics. Onset (derivative) was defined as day of the year of the maximum positive rate of increase in chlorophyll. The smoothed curve was first stabilised using a centred rolling mean (7‐day window) and the numerical derivative was computed; the onset of the seasonal bloom is the first sustained local maximum in that derivative (requiring ≥3 consecutive days of positive slope). Amplitude was defined as the difference between the seasonal peak chlorophyll and an early‐season baseline, where the baseline is the median chlorophyll in the first 30 days of the series (Nicholson et al., 2025). Smoothed chlorophyll time‐series alongside these metrics can be visualised for each region in Figures S4 and S5.

### 2.4 Data processing

All data processing and analysis was completed in R 4.5.2 (R Core Team, 2026). For our study of interannual variability in breeding success, we included burrows that had not been manipulated as part of experiments for other ongoing research (*n* = 766). However, many of the individuals included in the study had geolocators or were used in short‐term GPS deployments. To ensure biotelemetry device effects were not driving our results (Gillies et al., 2020), we ran a separate analysis excluding those with biotelemetry deployments (*n* = 376) and found results consistent with the full dataset (Figure S1).

We calculated geolocator position using twilight‐derived light thresholds through the geolight package (Lisovski & Hahn, 2012). These positions were first smoothed using a rolling 3 day mean, and a mean per day was then calculated to account for some of the error inherent in geolocator estimation (Liechti et al., 2018; Phillips et al., 2004). Phenology was estimated per individual using visual inspection of changes in longitude data over time (as in Siddiqi‐Davies, Wynn, Padget, Bond, et al., 2024; Siddiqi‐Davies, Wynn, Padget, Lewin, et al., 2024), as longitude is less prone to location error than latitude around the equinoxes. To determine the timing of arrival per individual at the breeding colony, we could not use the GLS light‐derived position data as there is insufficient spatial resolution to identify colony visits. Instead, we used the immersion logger data as nights with lower immersion have been validated to represent nocturnal colony visitation (Padget, 2017). Colony visits were determined using the mixtools package (Benaglia et al., 2009) to separate two distributions in the number of immersion events per night (Siddiqi‐Davies, Wynn, Padget, Lewin, et al., 2024), and nights with >50% probability of belonging to the drier peak were assigned as a colony visitation.

### 2.5 Data analysis

To determine whether previous breeding phenology influenced non‐breeding duration, we fitted a linear mixed‐effects model with non‐breeding duration (days) as the response variable and migration departure date in the previous breeding season (Y0) as a fixed effect, including year and pair identity as random effects (Model 11). Non‐breeding duration was defined as the number of days between post‐breeding migration departure and return to the breeding colony the following spring.

To test whether variation in non‐breeding duration influenced subsequent breeding phenology beyond persistent individual differences in breeding timing, we fitted a second linear mixed‐effects model with lay date (Y1) as the response variable (model 12). Non‐breeding duration was decomposed into an individual's mean non‐breeding duration and annual deviations from that mean, allowing us to separate persistent individual phenotypes from annual variation in non‐breeding duration. Migration departure date was also included as a fixed effect, allowing us to assess whether non‐breeding duration influenced subsequent lay date independently of when individuals departed. Year and pair identity were included as random effects.

We used the previous year's migration departure date because it has been found to be correlated with lay date in the same year (Siddiqi‐Davies, Wynn, Padget, Lewin, et al., 2024; Siddiqi‐Davies, Wynn, Padget, Bond, et al., 2024), allowing inclusion of a larger sample of geolocator‐tagged individuals for which previous breeding records were unavailable. However, we additionally conducted a supplementary analysis using the subset of individuals with lay date records in consecutive breeding seasons (*n* = 100), replacing migration departure date with the previous year's lay date (Model 25).

To understand whether individual differences in migratory destination influence the timing of breeding, we then investigated the effect of an individual's maximum overwinter foraging latitude (i.e. the most southerly migratory destination reached) averaged across years (Model 13), as well as annual deviations from this value, on lay date using a within–between (centred) mixed modelling approach (van de Pol & Wright, 2009). This approach allowed us to distinguish effects of consistent among‐individual differences in overwintering destination from effects of within‐individual inter‐annual variation in migratory destination.

As both pair members must be present before breeding can proceed, we expected the arrival date of the second‐returning partner to place the strongest constraint on lay date. However, the arrival date of the first returning partner may also influence laying if earlier arrival increases the time available for breeding preparation such as nest re‐establishment, defence and post‐migratory foraging. We therefore tested the separate effects of first‐ and second‐partner arrival dates on lay date. To understand whether lay date was associated with the arrival timing of either member of the breeding pair, we used the subset of pair‐years in which both partners had geolocator data available prior to laying (*n* = 63 individuals). For each pair‐year (*n* = 40), we identified the arrival date of the first returning partner and the arrival date of the second‐returning partner. We then fitted a linear mixed‐effects model with lay date as the Gaussian response variable and first‐arrival date and second‐arrival date as fixed effects, with year and pair identity included as random effects (Model 14). We conducted a supplementary analysis to investigate whether the sex of the second‐returning bird influenced the relationship between arrival timing and lay date by including interactions between sex and both first‐ and second‐arrival dates (Model 27). We also investigated whether the arrival interval between the first‐ and second‐returning birds influenced lay date, with the arrival date of the first‐returning bird included as an additional fixed effect (Model 28).

For breeding pairs in which immersion data were available for both partners prior to laying (*n* = 26 pairs), we additionally quantified pre‐laying colony overlap as: (i) the date of the first overlapping colony visit by both pair members, and (ii) the total number of overlapping colony visits prior to laying. We fitted a second linear mixed‐effects model with lay date as the Gaussian response variable and first overlapping visit date and total number of overlapping visits as fixed effects, again including year and pair identity as random intercepts (Model 15).

To investigate the effect of pair experience on lay date, we ran a model including the number of known years breeding together as a fixed effect, including pair ID as a random effect (Model 16). While we do not have records of when a partnership formed, as we have monitored burrows for 16 years within our study plot, we can assume that longer observed pairings will represent some of the signal for pair experience as birds begin breeding after 3–9 years and live until an average age of 15 (Brooke, 1990).

We applied generalised linear mixed‐effects models using the lme4 package (Bates et al., 2015) with a binomial response variable to investigate whether breeding success varied with the interactive fixed effects of lay date (day of the year) and year of study, including bird‐pair ID as a random effect (Model 1). To assess the robustness of this result and ensure a single year was not driving any temporal trends, we applied leave‐one‐out jack knifing where we excluded each year of observation and re‐ran the full model.

To test whether changes in chlorophyll phenology and amplitude were driving annual variation in the relationship between lay date and breeding success, we ran two models for both the Celtic (Models 2–3) and Irish sea (Models 4–5), swapping the interactive effect of year for the onset derivative and amplitude. As shearwaters that fail a breeding attempt typically do so during the egg phase or within the first few days after hatching, overall breeding success largely reflects early season environmental conditions. To assess a complementary metric of reproductive performance reflective of later breeding‐season conditions, we applied the same model structure to linear mixed‐effects models of chick peak mass (g) (models 6–10). Chick peak mass is typically reached within the final 2 weeks before fledging and reflects the intensity of adult chick provisioning. Significance was assessed using likelihood ratio estimation and mean effect sizes and 95% confidence intervals were calculated through bootstrapping 1000 simulations of fixed effects using the arm package (Gelman & Hill, 2006).

To assess whether population‐level breeding phenology tracked interannual variation in marine phenology, we fitted separate linear models relating mean annual lay date to annual bloom onset in the Irish and Celtic Seas (Models 19–20), with mean lay date calculated annually to match the temporal resolution of bloom onset. We also examined temporal trends in bloom phenology by modelling bloom onset and bloom amplitude as functions of year for each region (Models 21–24). To aid interpretation of bloom metrics and assess whether they represented independent environmental signals, we calculated Pearson correlation coefficients among bloom onset, bloom amplitude, and the coefficient of variation (CoV) of seasonal chlorophyll concentrations, both within and between the Irish and Celtic Seas.

To investigate whether lay date is shifting over time, we modelled the effect of year on lay date by splitting the effect into two components: the average year effect and deviations from the average year, with bird pair ID as a random effect (Model 17). This approach allowed us to distinguish between within‐individual effects (year‐to‐year variation for the same breeding pair) and between‐pair effects (variation across individuals), using the subject centring method (van de Pol & Wright, 2009). We additionally applied this method to assess whether associations between lay date and breeding success arose from within‐individual variation in timing or from consistent differences among individuals, including an interactive effect between within‐individual variation and year, and individual ID as a random effect (Model 18).

As these hypotheses (Table S1) rely on distinct datasets, they could not be combined into a single global model; instead, each tests a separate mechanism we predict may influence lay date. For all mixed‐effects models present in this analysis, *p* values were adjusted using the Benjamini and Hochberg method to control the false discovery rate per hypothesis (Benjamini & Hochberg, 1995). All continuous variables were centred to a mean of zero and standardised to a standard deviation of one prior to analysis.

## 3 RESULTS

### 3.1 Individual migratory phenology and behaviour

Migratory phenology exerted a strong effect on non‐breeding duration (Figure 1a), with birds departing later on migration having shorter non‐breeding periods (Model 11; *β* = −9.20, 95% CI [−10.84, −7.63], *χ* ^2^ = 87.64, *p* < 0.001). Separately, migration departure date significantly predicted lay date (Model 12; *β* = 1.67, 95% CI [0.61, 2.76], *χ* ^2^ = 7.91, *p* = 0.006), whereby individuals departing later in year Y0 laid later in year Y1.

**FIGURE 1:**
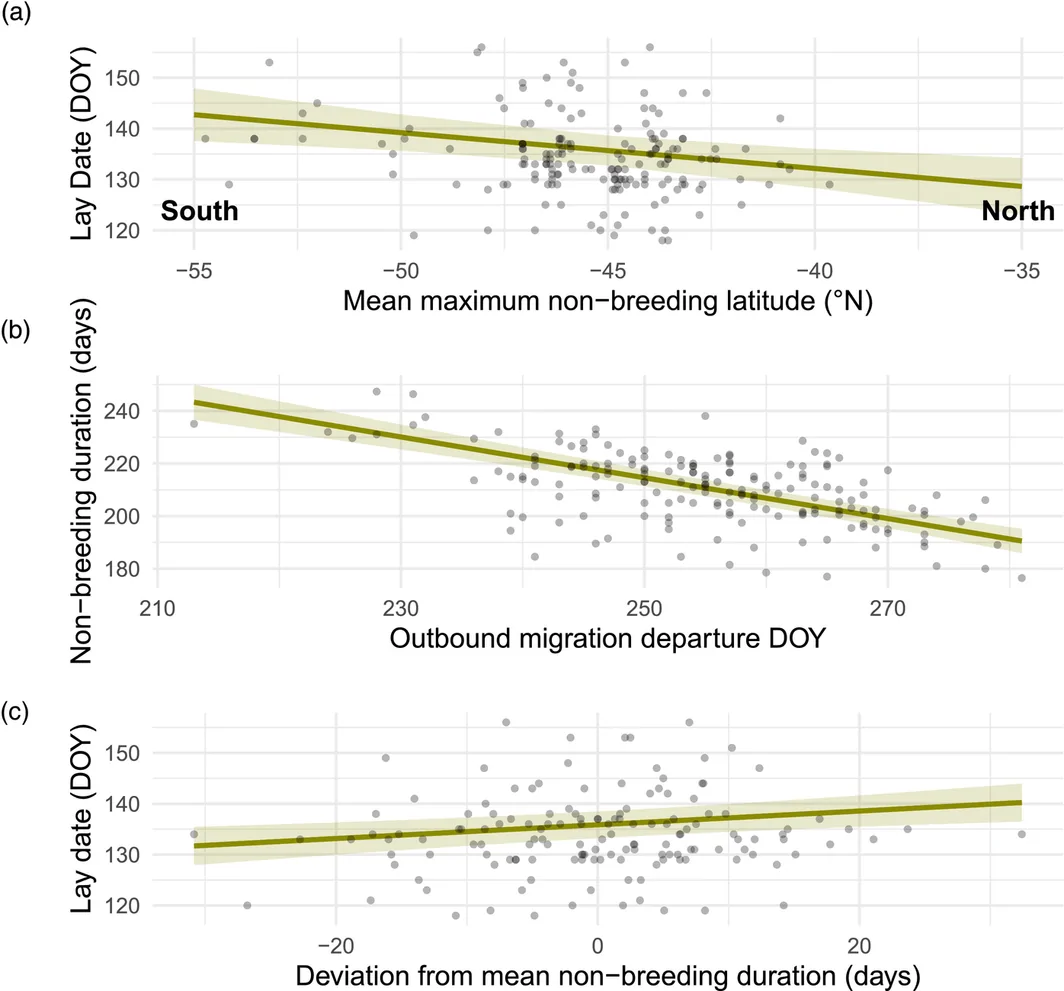
Effects of migratory behaviour on lay date. (a) Lay date versus mean maximum non‐breeding latitude (averaged across years per individual; Model 13). (b) The effect of outbound migration departure date on non‐breeding duration (time from outbound departure to homebound return; Model 11). (c) Lay date in relation to annual within‐individual deviations from the mean (averaged across years per individual) non‐breeding duration (Model 12). Trends and confidence intervals are from mixed‐effects models.

Mean non‐breeding duration was positively associated with lay date (Model 12; *β* = 2.69, 95% CI [1.52, 3.85], *χ* ^2^ = 15.71, *p* < 0.001), indicating that individuals that generally spent longer away from the breeding colony also tended to breed later. Annual deviations from an individual's mean non‐breeding duration were also positively associated with lay date (Model 12; *β* = 1.20, 95% CI [0.47, 1.94], *χ* ^2^ = 9.46, *p* = 0.003), indicating that individuals spending longer away than usual also laid later than expected. In combination, these results suggest that both persistent individual differences and annual variation in non‐breeding duration contribute to subsequent breeding phenology (Figure 1c).

In a supplementary analysis restricted to individuals with lay date records in consecutive years (*n* = 100), previous lay date significantly predicted lay date in the following breeding season (Table S5; Model 25), indicating repeatability in breeding phenology. However, previous lay date did not significantly predict non‐breeding duration (Table S5; Model 26). This suggests that while breeding phenology is repeatable among years, variation in non‐breeding duration is not simply a consequence of previous lay date.

Individuals that migrated to significantly more southerly latitudes when averaged across years had later lay dates (Model 13; *β* = −1.82, 95% CI [−2.89, −0.69], *χ* ^2^ = 9.50, *p* = 0.003), indicating that between‐individual repeatable strategies may influence lay date (Figure 1a). Meanwhile, annual deviations from the mean latitude did not affect lay date (Table S2; Model 13).

### 3.2 Pair synchrony

Pair synchrony also affected lay date, where lay date was influenced by the second‐returning birds' arrival date in a breeding pair (Model 14; *β* = 4.32, 95% CI [1.10, 7.58], *χ* ^2^ = 6.94, *p* = 0.01), whil the first returning bird had no significant effect on lay date (Figure 2a–c). Males and females were equally likely to return first (48% of first‐returning birds were female), and supplementary analyses found no evidence that the effects of first‐ or second‐arrival date on lay date differed according to sex (Model 27; Table S6). Additionally, longer intervals between the arrival dates of the first‐ and second‐returning birds were associated with later lay dates (Model 28; *β* = 2.36, 95% CI [0.58, 4.36], *χ* ^2^ = 7.06, *p* = 0.008; Table S6).

**FIGURE 2:**
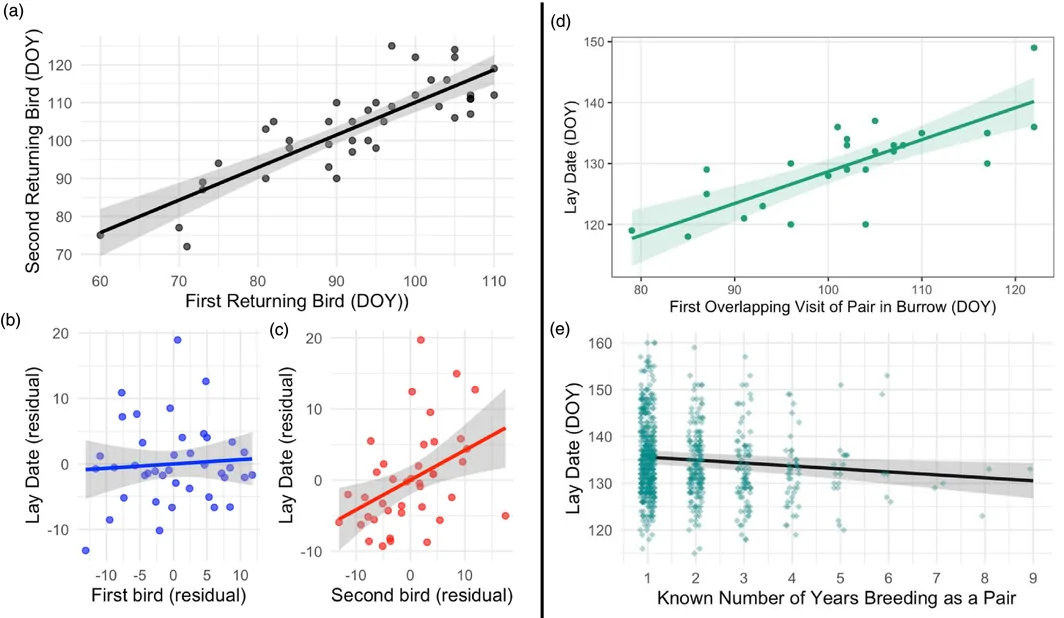
Effects of pair synchrony on lay date. (a) The correlation between the first and second‐returning birds return dates and (b, c) residuals from the relationship in (a) plotted against lay date to show the lack of effect on lay date of the first returning bird and the substantial effect of the second‐returning bird (Model 14). (d) First overlapping burrow visit (from immersion data) versus lay date (Model 15). (e) Known years breeding together versus lay date (Model 16). Trends and confidence intervals in (d, e) are from mixed‐effects models.

Lay date was additionally defined by the timing of the first overlapping colony visit between partners (Model 15; *β* = 5.90, 95% CI [4.00, 7.90], *χ* ^2^ = 25.66, *p* < 0.001) and not the total number of overlaps during the pre‐laying period, suggesting breeding phenology is partially constrained by pair coordination in re‐union at the colony (Figure 2d).

Pair experience additionally played a role in lay date, as indicated by the known number of years breeding as a pair, where birds known to be breeding for more years laid significantly earlier (Model 16; *β* = −0.74, 95% CI [−1.27, −0.18], *χ* ^2^ = 7.29, *p* = 0.01; Figure 2e).

### 3.3 Interannual variation in lay date and breeding success

Breeding success varied with lay date differently among years, with a significant interaction between lay date and year (Model 1; *β* = 0.27, 95% CI [0.05, 0.46], *χ* ^2^ = 7.27, *p* = 0.007). This indicates that the fitness consequences of laying early or late changed through time, with later laying becoming less costly in recent years (Figure 3). Leave‐one‐year‐out jack knifing confirmed the robustness of this result, with the interaction term remaining significant in all 16 iterations and bootstrapped estimates varying only modestly (0.23–0.32), while all 95% confidence intervals excluded zero.

**FIGURE 3:**
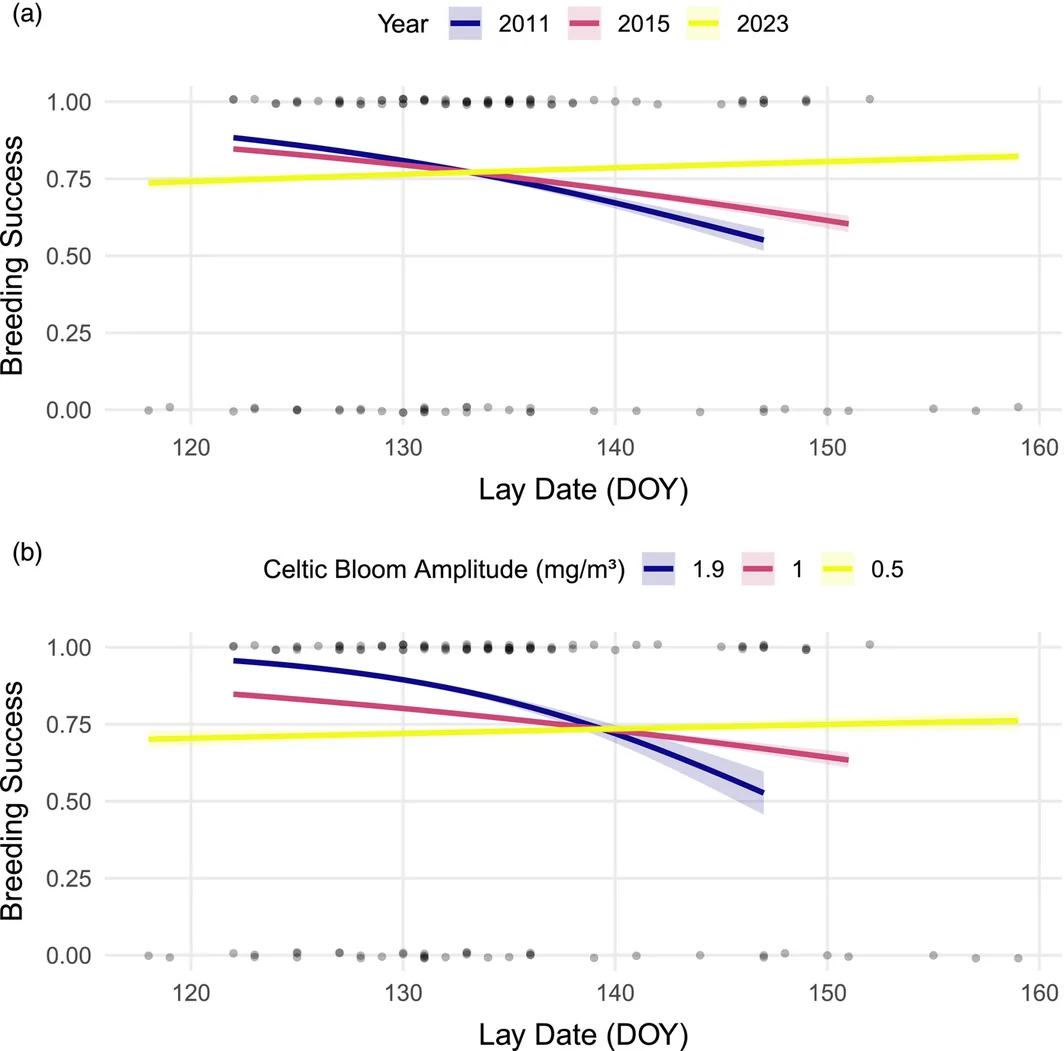
Effects of lay date on breeding success. (a) The effect of lay date on breeding success in relation to year (Model 1) and (b) Celtic Sea bloom amplitude (mg/m^3^) variation (Model 2). Colours align with the specific year of study (purple: 2011/1.9 mg/m^3^, red: 2015/1 mg/m^3^, yellow: 2023/1.9 mg/m^3^). All trends and confidence intervals are plotted from mixed‐effects model outputs. Three years with varying bloom Celtic Sea bloom amplitudes were selected to illustrate temporal variation in the relationship with breeding success and lay date. Relationships with lay date and breeding success for all 16 years of study can be visualised in Figure S2.

Chick peak mass also showed a significant interaction between lay date and year (Model 6; *β* = 7.61, 95% CI [1.04, 14.48], *χ* ^2^ = 4.81, *p* = 0.03). This indicates that the relationship between lay date and chick peak mass varied among years, with later lay dates associated with higher peak chick mass in recent years (Figure 4). Leave‐one‐year‐out jack knifing confirmed the sign and magnitude of the interaction, with estimates remaining positive in all iterations (4.9–10.3) and statistically significant in 10 of 13 runs (LRT *p* < 0.05), indicating that the result was not driven by a single anomalous year. Relationships between lay date and reproductive performance for individual years are shown in Figures S2 and S3.

**FIGURE 4:**
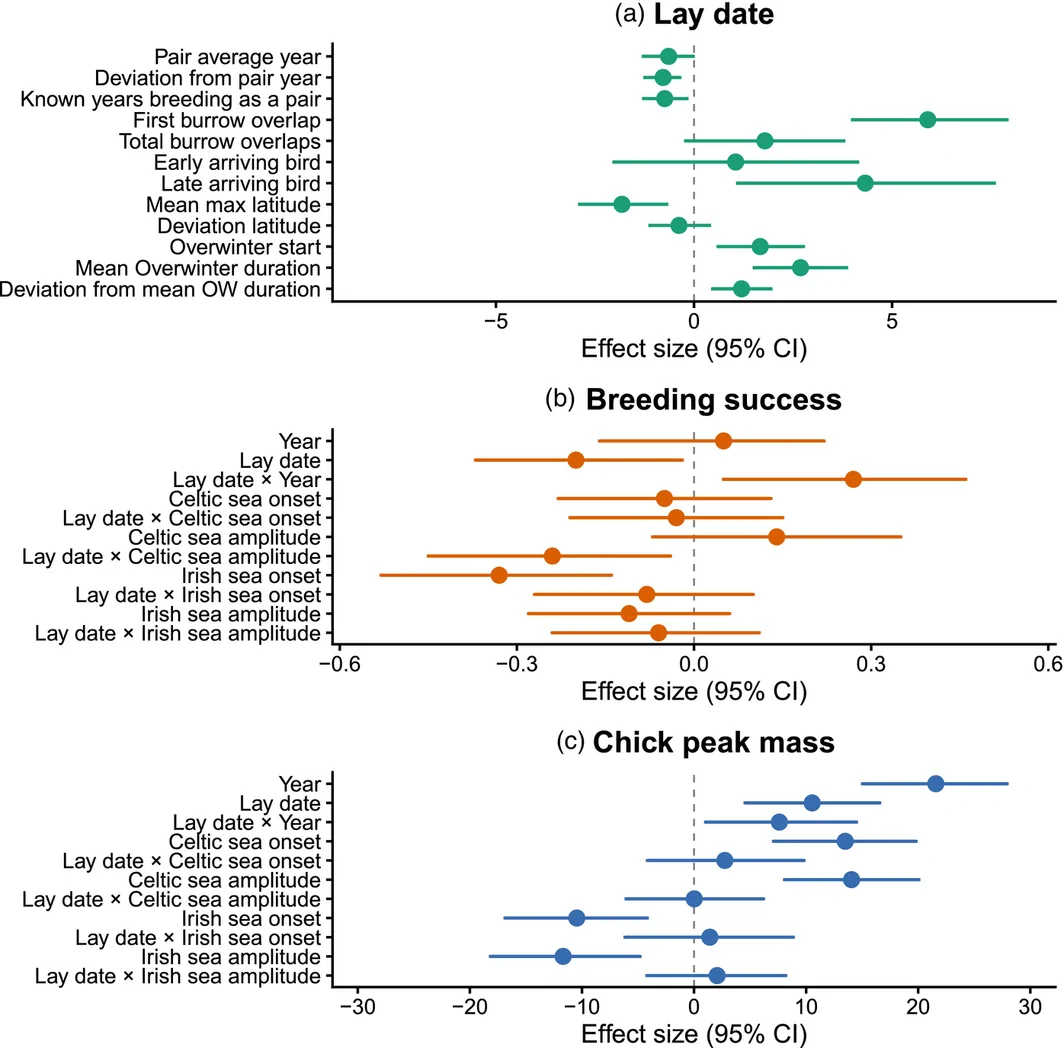
Effect sizes (±95% confidence intervals) of scaled predictor variables on lay date, breeding success, and chick peak mass. Confidence intervals that do not overlap zero represent statistically supported effects.

To distinguish between‐individual and within‐individual effects of lay date on breeding success, we decomposed lay date into an individual's mean lay date and annual deviations from that mean. Mean lay date was negatively associated with breeding success (Model 18; *β* = −0.16, 95% CI [−0.28, −0.03], *χ* ^2^ = 5.80, *p* = 0.02), indicating that individuals breeding earlier on average achieved greater breeding success. Annual deviations from an individual's mean lay date were not associated with breeding success overall (Model 18; *β* = −0.07, 95% CI [−0.18, 0.04], *χ* ^2^ = 1.47, *p* > 0.05), but interacted significantly with year (Model 18; *β* = 0.16, 95% CI [0.02, 0.29], *χ* ^2^ = 5.14, *p* = 0.02), indicating that the fitness consequences of breeding earlier or later than an individual's average lay date varied through time.

### 3.4 Irish and Celtic Sea bloom onset and amplitude

Bloom onset generally occurred during the pre‐laying and early breeding period, overlapping with the period between colony return and laying, although onset timing varied among years and between regions. Irish Sea bloom onset occurred on average 20 days before mean lay date, ranging from 51 days before to 12 days after mean lay date. Celtic Sea bloom onset occurred on average 37 days before mean lay date, ranging from 16 to 56 days before mean lay date. Mean annual lay date increased significantly with Irish Sea bloom onset (Model 20; *β* = 0.08, 95% CI [0.00, 0.15], SE = 0.04, *t* = 2.16, *p* = 0.049), indicating that birds laid later in years with later bloom onset. A similar positive relationship was observed for Celtic Sea bloom onset, although confidence intervals overlapped zero (Model 19; *β* = 0.14, 95% CI [−0.02, 0.29], SE = 0.07, *t* = 1.90, *p* = 0.07). These results suggest that breeding phenology broadly tracked interannual variation in marine bloom timing.

Bloom metrics were only weakly correlated between the Irish and Celtic Seas (onset: *r* = 0.23; amplitude: *r* = 0.10), indicating that the two regions represented largely independent environmental signals (Figure S6). Within the Irish Sea, bloom onset and amplitude were moderately positively correlated (*r* = 0.55), whereas no relationship was evident in the Celtic Sea (*r* = −0.10). Bloom amplitude was positively correlated with the coefficient of variation (CoV) of seasonal chlorophyll concentrations in both regions, particularly in the Celtic Sea (*r* = 0.85; Irish Sea: *r* = 0.54), indicating that years with stronger blooms were also characterised by greater temporal variability in chlorophyll concentrations across the season.

We found no evidence that Celtic Sea bloom onset affected breeding success or altered the relationship between lay date and breeding success (Table S2; Model 3). However, the annual amplitude of the Celtic Sea bloom significantly interacted with lay date (Model 2; *β* = −0.24, 95% CI [−0.45, −0.04], *χ* ^2^ = 5.11, *p* = 0.04), indicating that birds were increasingly penalised for laying late in years with stronger blooms (Figure 3b).

In the Irish Sea onset model (Table S2; Model 5), bloom onset did not significantly interact with lay date, but later bloom onset was associated with lower breeding success (*β* = −0.33, 95% CI [−0.53, −0.14], *χ* ^2^ = 10.77, *p* = 0.001). We found no significant effects of Irish Sea bloom amplitude on breeding success (Table S2; Model 4).

For chick peak mass, we found no evidence that bloom metrics altered the relationship between lay date and chick peak mass, as none of the lay date × bloom metric interactions were significant in either the Celtic or Irish Sea models. However, in the Celtic Sea onset model, later bloom onset was associated with higher chick peak mass (Model 8; *β* = 13.49, 95% CI [7.09, 19.80], *χ* ^2^ = 17.39, *p* < 0.001). Similarly, in the Celtic Sea amplitude model, stronger blooms were associated with higher chick peak mass (Model 7; *β* = 14.05, 95% CI [8.08, 20.05], *χ* ^2^ = 19.74, *p* < 0.001).

In contrast, in the Irish Sea onset model (Model 10), later bloom onset was associated with lower chick peak mass (*β* = −10.46, 95% CI [−16.85, −4.18], *χ* ^2^ = 8.72, *p* = 0.03), while stronger Irish Sea blooms were also associated with lower chick peak mass in the Irish Sea amplitude model (Model 9; *β* = −11.68, 95% CI [−18.16, −4.82], *χ* ^2^ = 12.14, *p* < 0.001). Later or stronger Celtic Sea blooms therefore seem to predict an increase in chick peak mass independent of lay date, while the opposite pattern occurs in the Irish Sea, with later, stronger Irish Sea blooms being correlated with lower chick peak mass (Figure 4).

### 3.5 Temporal trends in lay date

Lay date showed a significant advance over time at the population level, with pairs breeding in later calendar years laying slightly earlier on average (Model 17; *β* = −0.64, 95% CI [−1.28, −0.02], *χ* ^2^ = 4.00, *p* = 0.02). In addition, within pairs, inter‐annual deviations from a pair's mean breeding year were associated with substantially earlier lay dates (Model 17; *β* = −0.78, 95% CI [−1.24, −0.36], *χ* ^2^ = 12.61, *p* < 0.001), indicating a consistent within‐pair advancement in lay date over time.

We did not detect any significant temporal trends in bloom onset that could explain this advancement (Table S4). However, Celtic Sea bloom amplitude declined significantly over the study period (Model 21; *β* = −0.04, 95% CI [−0.07, −0.01], SE = 0.02, *t* = −2.50, *p* = 0.02).

## 4 DISCUSSION

Although earlier laying generally resulted in higher reproductive success, our results suggest that there is unlikely to be a single optimal breeding date for all individuals and all years. Decomposing lay date into within‐ and between‐individual components revealed that individuals breeding earlier on average achieved greater reproductive success, whereas annual deviations from an individual's average lay date were not consistently associated with breeding success. This suggests that early breeding may partly reflect differences in individual quality (Arnold et al., 2006; Bolton, 1991), with higher quality individuals more likely to breed early, rather than early laying consistently providing an inherent reproductive advantage in all years (Table S3).

However, we also found substantial interannual variability in the relationship between lay date and breeding success, whereby laying later appears to be less costly, or even beneficial in recent years (Figure 3). This suggests that there is some selection on breeding phenology itself, though it is not constant through time, and may weaken or change direction when conditions change (Morrissey & Hadfield, 2012). Such temporal variation in selection has been observed in other species and linked to environmental factors and alignment with prey phenology (Sheldon et al., 2003; Visser et al., 2021). For example, earlier laying has been shown to be more favoured in years with warmer spring arrival temperatures (Visser et al., 2015), or where there were smaller intervals between prey and breeding phenology (Charmantier et al., 2008). Meanwhile, in some species, the strength of selection was shown to vary among years without systematic covariation with measured environmental quality (Reed et al., 2009; Shave et al., 2019).

Although many seabirds have not exhibited phenological changes in response to climate change (Keogan et al., 2018), in our study, we detect a slight advancement in lay dates, where within‐breeding pairs, birds advance their lay date by half a day per year (Table S2). This could represent an adaptive response to environmental change (Gienapp et al., 2013; Grémillet & Boulinier, 2009; Sauve et al., 2019). A weakening of selection for early laying in recent years could indicate that birds have generally become better aligned with the environmental conditions that favour successful breeding. We explored temporal shifts in bloom phenology as a potential driver, consistent with studies that link breeding timing to peak food availability (Van Noordwijk et al., 1995). Although population‐level lay date weakly tracked interannual variation in bloom phenology, we did not detect directional trends over time in bloom onset, nor significant interactions between bloom timing and lay date on breeding success. This suggests that changing bloom onset is unlikely to explain the temporal change in selection on lay date.

We did however find that years with stronger Celtic Sea blooms in phytoplankton selected for early laying and that bloom amplitude declined over time (Clausen & Clausen, 2013). Although the strength of the Celtic Sea bloom is not an indicator of resource timings, this result suggests that other components of the seasonal phytoplankton bloom may influence the relationship between breeding phenology and reproductive success (Tremblay et al., 2003). We found that years with stronger blooms were also characterised by more temporally concentrated productivity. Consequently, the observed increase in selection for earlier laying during strong bloom years may reflect a narrower seasonal window of resource availability (Visser et al., 2006). While it is difficult to disentangle complex pathways linking primary production to predator breeding success, our results suggest that both variation in individual quality and temporal fluctuations in selection contribute to the maintenance of variation in breeding phenology within the population.

We also detected contrasting effects of phytoplankton timings and availability in different oceanic areas within the breeding season foraging range. Later Irish Sea bloom onset reduced breeding success, and later or stronger Irish Sea blooms were associated with lower chick peak mass, suggesting that Irish Sea phenology may constrain reproductive output (Figure 4). In contrast, stronger or later Celtic Sea blooms were associated with increased chick peak mass, despite stronger Celtic Sea blooms also increasing the cost of laying late. This contrast may reflect stage‐specific use of different foraging areas: chick‐provisioning adults tend to forage more locally than during other breeding stages, including within the Celtic Sea, and may therefore benefit from a later or stronger seasonal bloom that aligns more closely with chick‐rearing foraging effort (Dean et al., 2013). However, this does not explain why later Irish Sea phenology causes declines to both breeding success and chick peak mass. An alternative explanation may be that the species composition of prey may vary spatially, whereby later or stronger phytoplankton blooms represent different prey communities between the Celtic and Irish Sea (Burthe et al., 2012; Edwards & Richardson, 2004). Therefore, later or stronger Irish Sea blooms may result in prey communities that are suboptimal for shearwater self and chick provisioning.

While changes in patterns of primary productivity can have ecosystem‐wide effects, the translation of bloom metrics into meaningful shifts in prey availability is complex and likely region‐specific, requiring detailed sampling of prey communities (Grémillet et al., 2008). Therefore, although our results provide some evidence for significant effects of spring bloom characteristics, they require further investigation to fully resolve the trophic pathways linking phytoplankton phenology to reproductive outcomes (Nowicki et al., 2023). As we focus on breeding success as one component of fitness, we cannot quantify total selection on lay date; trade‐offs with other fitness components, such as adult survival or future reproduction, may also influence the optimal timing of breeding. Nonetheless, our findings support that selection for earlier breeding phenology can vary and thus may help explain the persistence of substantial lay date variation within a population. Such variation may inadvertently buffer populations against climatic changes (Reed et al., 2013), particularly where environmental effects on prey communities are complex and non‐linear. This may be especially important in organisms with extreme life‐history strategies such as seabirds, which typically produce a single offspring per year and invest heavily in reproduction (Elliott et al., 2021; Hemmings & Birkhead, 2020).

We found that individuals departing later on their post‐breeding migration tended to have shorter non‐breeding periods, presumably to compensate for delayed departure (Siddiqi‐Davies, Wynn, Padget, Bond, et al., 2024). Individuals that generally spent less time away from the breeding colony or departed earlier on migration bred earlier, suggesting repeatable differences in breeding phenology. However, annual deviations from an individual's typical non‐breeding duration also influenced breeding timing (Figure 1). The length of the non‐breeding period may reflect both individual quality and environmental conditions experienced during migration and over winter (Acerenza, 2016; Rotics et al., 2018). Birds in better condition may need less time to recover following reproduction and have increased efficiency over migration and overwintering self‐provisioning (Desprez et al., 2018; Dittmann & Becker, 2003), whereas storms (Laurenson et al., 2025), anomalous temperatures (Remisiewicz & Underhill, 2022; Visser et al., 2015), or other adverse conditions may delay return and subsequent breeding. Our results indicate that plasticity in breeding timing is influenced by both repeatable individual differences and annual variation in non‐breeding duration (Fayet et al., 2016; Gatt et al., 2020; Norris & Taylor, 2006).

We additionally found that individuals that generally migrated to less southerly destinations laid earlier (Figure 1). Given that shearwaters travel thousands of kilometres annually at high speeds, it is unlikely that this effect is driven solely by increased migratory distance delaying return and subsequent breeding, particularly as year‐to‐year deviations from an individual's mean latitude had no significant effect on lay date. These annual deviations from their typical migratory destination may reflect shifts in response to environmental oscillations in resource distribution via the El Niño‐Southern Oscillation (Siddiqi‐Davies, Wynn, Padget, Lewin, et al., 2024). We therefore suggest that individuals that generally winter at less southerly destinations may be more efficient foragers and do not need to travel as far to access suitable foraging opportunities, such that the observed relationship between latitude and breeding phenology may be more indicative of individual condition than of environmental differences (Descamps et al., 2011).

We also find that lay date is not determined solely by individual behaviour, and synchrony between breeding pairs plays a role. Specifically, the second‐returning birds' arrival date limits advancement in lay date, regardless of how early the first returning bird arrives at the breeding colony (Figure 2). We also find, consistent with previous research, that lay date for this species advances with increased pair experience (Brooke, 1978, 1990). These results highlight that for species that exhibit long‐duration partnerships, plasticity in breeding phenology may be limited by their partner's behaviour. This may be an important factor to consider when examining differences in advancements in breeding phenology with climatic changes between avian species, even within similar environments (Gordo, 2007).

To conclude, we find that breeding earlier does not always equate to higher reproductive success, and that lay date reflects the combined influence of persistent individual differences, annual variation in non‐breeding duration, and pair synchrony and experience. Although based on a single species, our findings highlight two broader considerations for understanding phenological responses to environmental change. First, well‐documented relationships between breeding timing and reproductive success cannot necessarily be assumed to remain constant through time. Second, phenological plasticity may be constrained not only by individual migratory phenotypes, but also by the behaviour and timing of breeding partners. This may be particularly relevant for long‐lived species with persistent pair bonds, in which breeding decisions depend on the coordination of two individuals. Comparative studies across taxa that differ in pair duration could therefore help establish when pair behaviour can facilitate or constrain phenological responses to environmental change. Finally, although phytoplankton phenology provides a proxy for energy input into a system, future work should also aim to disentangle how prey quality and quantity within different foraging areas impact reproductive success, and how long‐term breeding pairings may limit phenological advancement in long‐lived species.

## AUTHOR CONTRIBUTIONS

Katrina Siddiqi‐Davies conceived the study, analysed the data and led the writing. Katrina Siddiqi‐Davies and Natasha Gillies developed the study design. Tim Guilford designed the methodology and managed the project. Katrina Siddiqi‐Davies, Natasha Gillies, Hector Bailey, Sarah Bond, Annette Fayet, Holly Kirk, Lewis Lancaster‐Reeves, Joe Morford, Oliver Padget, Akiko Shoji, Martyna Syposz, Joe Wynn and Tim Guilford collected data, revised the manuscript and approved the final version.

## CONFLICT OF INTEREST STATEMENT

The authors declare no conflicts of interest.

## STATEMENT ON INCLUSION

This study was conducted in Wales and includes local researchers alongside collaborators based internationally. The researchers also provided a Welsh‐language summary (abstract) of the study, translated by Catherine Burns, as they wanted to recognise and celebrate the Welsh cultural heritage of Skomer Island (Ynys Sgomer).

## Supporting information


**Table S1.** Summary of mixed‐effects models investigating the causes and consequences of lay date variation under each hypothesis. For each model, the response variable, predictors, significant terms (with *p* values corrected using the Benjamini–Hochberg procedure within each hypothesis), and a brief interpretation are shown. Full model outputs, including coefficients and confidence intervals, are provided in Table S2.
**Table S2.** Summary of all mixed‐effects models used in the analysis, showing coefficients, sample sizes, bootstrapped 95% confidence intervals, effect sizes and p values calculated from *χ* ^2^ likelihood ratio tests. Significant results are highlighted in bold, and levels of statistical significance are indicated by asterisks (*p* < 0.05*, *p* < 0.01**, *p* < 0.001***).
**Table S3.** Results of a within‐subject centring (van de Pol & Wright, 2009) decomposition model used to distinguish between‐individual and within‐individual effects of laying date on breeding success. The model included each individual's mean laying date (between‐individual effect) and annual deviations from that mean (within‐individual effect). Shown are model coefficients, sample sizes, bootstrapped 95% confidence intervals, effect sizes, and *p*‐values derived from likelihood ratio tests (*χ* ^2^). Significant results are highlighted in bold; levels of statistical significance are indicated by asterisks (*p* < 0.05*, *p* < 0.01**, *p* < 0.001***).
**Table S4.** Results of linear models testing whether annual mean laying date tracked interannual variation in phytoplankton bloom onset, and whether bloom onset and bloom amplitude changed over the study period in the Irish and Celtic Seas.
**Table S5.** Supplementary analysis for the subset of individuals with laying date records in consecutive breeding seasons (*n* = 100), examining the effects of previous year's laying date on non‐breeding duration and subsequent laying date. This analysis was conducted as post‐breeding departure date and laying date are correlated but not equivalent measures, allowing assessment of whether observed migration–breeding relationships could reflect repeatability in breeding phenology. Shown are model coefficients, sample sizes, bootstrapped 95% confidence intervals, effect sizes, and *p*‐values derived from likelihood ratio tests (*χ* ^2^). Significant results are highlighted in bold; levels of statistical significance are indicated by asterisks (*p* < 0.05*, *p* < 0.01**, *p* < 0.001***).
**Table S6.** Supplementary analyses testing whether the relationships between first‐ and second‐arrival dates and lay date differed according to the sex of the second‐returning bird, and whether the arrival interval between pair members influenced lay date as a measure of within‐pair arrival synchrony. Shown are model coefficients, sample sizes, bootstrapped 95% confidence intervals, effect sizes and *p*‐values derived from likelihood ratio tests (*χ* ^2^). Significant results are highlighted in bold; levels of statistical significance are indicated by asterisks (*p* < 0.05*, *p* < 0.01**, *p* < 0.001***).
**Figure S1.** Sensitivity analysis of the effect of lay date on breeding success. Points show coefficient estimates (±95% CI) from mixed‐effects models fitted to the full dataset (*n* = 766) and to untracked birds only (*n* = 376). Although the effects are no longer statistically significant when restricted to untracked birds, the direction and magnitude of the estimate are consistent, indicating reduced power rather than an effect of tracking devices.
**Figure S2.** Modelled relationships between breeding success and lay date plotted for each year of observation. Panels are shown to support mixed‐effects model outputs and to illustrate interannual variability in the relationship between breeding success and lay date.
**Figure S3.** Modelled relationships between chick peak mass and lay date plotted for each year of observation. Panels are shown to support mixed‐effects model outputs and to illustrate interannual variability in the relationship between breeding success and lay date.
**Figure S4.** Annual chlorophyll phenology metrics for the Celtic Sea. Smoothed (LOESS: span = 0.25) daily chlorophyll time series are shown for each year. Dashed vertical lines indicate the timing of the derivative‐based bloom onset, and seasonal amplitude values are displayed in each panel. Metrics were calculated independently for each year.
**Figure S5.** Annual chlorophyll phenology metrics for the Irish Sea. Smoothed (LOESS: span = 0.25) daily chlorophyll time series are shown for each year. Dashed vertical lines indicate the timing of the derivative‐based bloom onset, and seasonal amplitude values are displayed in each panel. Metrics were calculated independently for each year.
**Figure S6.** Spatial distribution of Manx shearwater foraging locations during incubation (blue) and chick‐rearing (red) periods. Points represent a random subsample of GPS positions used to illustrate broad‐scale foraging distributions. Shaded polygons indicate the Celtic Sea (green) and Irish Sea (blue) regions used for phytoplankton bloom analyses. Foraging locations occurred within both regions during both breeding stages, although the relative distribution of locations differed between incubation and chick‐rearing.

## Data Availability

Data are available at zenedo https://doi.org/10.5281/zenodo.18744234 (Siddiqi‐Davies et al., 2026).
