# Supplementary material for: Why don't all birds lay early? Causes and consequences of breeding timing for a long‐lived migratory seabird
Source: J Anim Ecol. 2026 Oct 10;95(11):2347–61. doi: 10.1111/1365-2656.70363 (PMC13731836; doi:10.1111/1365-2656.70363)
Supplement: Supplementary file 1 [file JANE-95-2347-s001.docx]

**Supplementary Materials:**

**Table S1.** Summary of mixed‐effects models investigating the causes and consequences of lay date variation under each hypothesis. For each model, the response variable, predictors, significant terms (with p values corrected using the Benjamini–Hochberg procedure within each hypothesis), and a brief interpretation are shown. Full model outputs, including coefficients and confidence intervals, are provided in Supplementary Table S2.

| Hypothesis |  | Response | Predictors tested | Significant terms | Interpretation | | |
| --- | --- | --- | --- | --- | --- | --- | --- |
| H1.1 Lay date affects breeding success | Mod 1 | Success (binomial) | Lay date × Year | Interaction (p < 0.05)  Lay date (p<0.05) | Laying late reduces breeding success, but this effect is reduced in recent years. | | |
| H1.2 Bloom metrics (Celtic Sea) | Mod 2 | Success  (binomial) | Lay date × Amplitude | Interaction (p < 0.05)  Lay date (p<0.05) | Strong Celtic blooms increase the cost of laying late. | | |
|  | Mod 3 | Success  (binomial) | Lay date × Onset | NS | Onset not detected as a driver here. | | |
| H1.3 Bloom metrics (Irish Sea) | Mod 4 | Success  (binomial) | Lay date × Amplitude | NS | Amplitude not detected as a driver here | | |
|  | Mod 5 | Success  (binomial) | Lay date × Onset | Onset (p < 0.05) | Later Irish blooms reduce overall success. | | |
| H2.1 Lay date affects chick mass | Mod 6 | Peak chick mass (g) | Lay date × Year | Interaction (p < 0.01)  Year (p<0.001) Lay date (p<0.01) | Laying late has increased chick peak mass in recent years | | |
| H2.2 Bloom metrics (Celtic Sea) | Mod 7 | Peak chick mass (g) | Lay date × Amplitude | Amplitude (p<0.001)  Lay date (p<0.05) | Strong Celtic blooms increase peak mass (with  no interactive effect). | | |
|  | Mod 8 | Peak chick mass (g) | Lay date× Onset | Onset (p<0.01) | Later Celtic blooms increase chick peak mass  (with no interactive effect). | | |
| H2.3 Bloom metrics (Irish Sea) | Mod 9 | Peak chick mass (g) | Lay date × Amplitude | Amplitude (p<0.001) | Strong Irish blooms reduce chick peak mass  (with no interactive effect) | | |
|  | Mod 10 | Peak chick mass (g) | Lay date × Onset | Onset (p<0.01) Lay date (p<0.05) | Later Irish blooms reduce chick peak mass  (with no interactive effect) | | |
| H3. Migratory phenology and destination predicts lay date | Mod  11 | Non-breeding duration | Outbound migration start date | Start date (p<0.001) | Birds that set off later on migration have shorter non-breeding periods. | |  |
|  | Mod 12 | Lay date | Mean non-breeding duration + deviation + start date | Mean Duration (p<0.001)  Deviation (p<0.001)  Start date (p<0.001) | Earlier migration departure and shorter non-breeding duration predicted earlier laying | |  |
|  | Mod 13 | Lay date | Mean non-breeding minimum latitude + deviation from mean latitude | Mean minimum latitude (p<0.01) | Individuals that generally migrate to latitudes further south lay later |  |  |
| H4. Pair synchrony predicts lay date | Mod 14 | Lay date | First returning birds date+ second returning birds date | Second returning birds date (p<0.05) | The second returning bird predicts lay date |  |  |
|  | Mod 15 | Lay date | First date overlapping in the colony + total number of overlaps in the colony | First date overlapping in the colony (p<0.001) | The first date a pair re-unite at the colony after migrating predicts lay date |  |  |
|  | Mod 16 | Lay date | Known number of years breeding as a pair | Known years breeding as a pair (p<0.05) | Increased pair experience results in earlier lay dates |  |  |
| H5. Lay date is shifting over time | Mod 17 | Lay date | Average year+ deviations from the average year | Average year (p<0.05)  Deviation from average year (p<0.001) | Lay date shows slight advancement in recent years both within and between burrow pairs |  |  |

**Table S2.** Summary of all mixed‐effects models used in the analysis, showing coefficients, sample sizes, bootstrapped 95% confidence intervals, effect sizes, and p values calculated from χ² likelihood ratio tests. Significant results are highlighted in bold, and levels of statistical significance are indicated by asterisks (p < 0.05*, p < 0.01**, p < 0.001***).

| Response | Coefficient | | Effect Size | | Confidence Intervals | | *χ^2^* | | | p value | |
| --- | --- | --- | --- | --- | --- | --- | --- | --- | --- | --- | --- |
| Breeding Success (n=766)  Mod 1 | *Intercept*  **Lay Date (scaled, SD=7.95 days)**  Year (scaled, SD=4.92y)  **Interactive term (scaled)** | | 1.18  -0.20  0.05  0.27 | | 0.98, 1.38  -0.37, -0.02  -0.16, 0.22  0.05, 0.46 | | -  3.97  0.23  7.27 | | - 0.046*  >0.05  0.007** | | |
| Mod 2 | *Intercept*  **Lay Date (scaled, SD=7.95 days)**  Celtic Amplitude (scaled, SD=0.35 mg/m^3^)  **Interactive term (scaled)** | | 1.15  -0.22  0.14  -0.24 | | 0.95, 1.35  -0.41, -0.03  -0.07, 0.35  -0.45, -0.04 | | -  5.16  2.20  0.11 | | - 0.02*  >0.05  0.04* | | |
| Mod 3 | *Intercept*  Lay Date (scaled, SD=7.95 days)  Celtic Onset (scaled, SD= 9.32 days)  Interactive term (scaled) | | 1.14  -0.17  -0.05  -0.03 | | 0.96, 1.33  -0.34, 0.00  -0.23, 0.13  -0.21, 0.15 | | -  3.15  0.28  0.11 | | - >0.05  >0.05  >0.05 | | |
| Mod 4 | *Intercept*  Lay Date (scaled, SD=7.95 days)  Irish Amplitude (scaled, SD=0 .48 mg/m^3^)  Interactive term (scaled) | | 1.14  -0.18  -0.11  -0.06 | | 0.95, 1.33  -0.36, 0.01  -0.28, 0.06  -0.24, 0.11 | | -  3.68  1.60  0.41 | | - >0.05  >0.05  >0.05 | | |
| Mod 5 | *Intercept*  Lay Date (scaled, SD=7.95 days)  **Irish Onset (scaled, SD= 17.63 days)**  Interactive term (scaled) | | 1.20  -0.08  -0.33  -0.08 | | 0.99, 1.41  -0.27, 0.11  -0.53, -0.14  -0.27, 0.10 | | -  0.64  10.77  0.59 | | - >0.05  0.001**  >0.05 | | |
| Chick peak mass (g) (n=306)   Mod 6 | *Intercept*  **Lay Date (scaled, SD=7.61 days)**  **Year (scaled, SD=4.11y)**  **Interactive term (scaled)** | | 573.57  10.54  21.57  7.61 | | 566.92, 579.43  4.56, 16.56  15.04, 27.92  1.04, 14.48 | | - 10.46  40.80  4.81 | | -  0.0012**  <0.001*** 0.028* | | |
| Mod 7 | *Intercept*  **Lay Date (scaled, SD=7.61 days)**  **Celtic Amplitude (scaled, SD=0.19 mg/m^3^)**  Interactive term (scaled) | | 572.28  6.68  14.05  0.02 | | 565.53, 578.70  -0.04, 13.38  8.08, 20.05  -6.06, 6.20 | | -  4.12  19.74  0 | | -  0.04*  <0.001***  >0.05 | | |
| Mod 8 | *Intercept*  Lay Date (scaled, SD=7.61 days)  **Celtic Onset (scaled, SD= 9.04 days)**  Interactive term (scaled) | | 572.10  4.56  13.49  2.75 | | 565.29, 578.26  -1.89, 10.90  7.09, 19.80  -4.16, 9.80 | | -  1.90  17.39  0.61 | | -  >0.05  <0.001***  >0.05 | | |
| Mod 9 | *Intercept*  Lay Date (scaled, SD=7.61 days)  **Irish Amplitude (scaled, SD=0 .37mg/m^3^)**  Interactive term (scaled) | | 572.64  5.07  -11.68  2.06 | | 566.20, 579.38  -1.39, 11.64  -18.16, -4.82  -4.20, 8.18 | | -  2.44  12.14  0.45 | | -  >0.05  <0.001***  >0.05 | | |
| Mod 10 | *Intercept*  **Lay Date (scaled, SD=7.61 days)**  **Irish Onset (scaled, SD= 18.62 days)**  Interactive term (scaled) | | 572.64  9.96  -10.46  1.41 | | 565.38, 579.58  3.33, 16.70  -16.85, -4.18  -6.16, 8.85 | | -  7.42  8.72  0.16 | | -  0.006**  0.03**  >0.05 | | |
| Over-wintering duration  (n=173)   Mod 11 | *Intercept* **Over-wintering start date (scaled, SD=11.90)** | | 210.66 -9.20 | | 207.37, 213.84 -10.84, -7.63 | | -  87.64 | | -  <0.001*** | | |
| Lay date  (n=173)     Mod 12 | *Intercept*  **Mean over-wintering duration (scaled, SD=10.45)**  **Deviations from mean over-wintering duration (scaled, SD=8.99)**  **Over-wintering start date (scaled, SD=11.90)** | | 136.27  2.69  1.20  1.67 | | 133.45, 138.81  1.52, 3.85  0.47, 1.94  0.61, 2.76 | | -  15.71  9.46   7.91 | | - <0.001***  0.003**  0.006** | | |
| Lay Date  (n=169)    Mod 13 | *Intercept*  **Averaged Maximum latitude (° N) (scaled, SD = 2.58)**  Deviation from mean latitude (° N) (scaled, SD= 1.30) | | 136.11  -1.82  -0.38 | | 132.98, 139.20  -2.89, -0.69  -1.11, 0.39 | | -  9.50  0.93 | | -  0.003**  >0.05 | | |
| Lay Date (n=40)    Mod 14 | *Intercept*  **Late Bird Arrival Date (DOY) (scaled, SD= 12.50)**  Early Bird Arrival Date (DOY)  (scaled, SD = 12.77) | | 135.32  4.32  1.05 | | 131.71, 139.04  1.10, 7.58 -2.02, 4.13 | | -  6.94  0.34 | | -  0.01 *  >0.05 | | |
| Lay date  (n=26)     Mod 15 | *Intercept*  **Date of first overlap between partners in burrow (DOY)** (scaled, SD= 3.69)  Total number of overlaps in burrow(scaled, SD= 11.30) | | 130.00  5.90  1.79 | | 128.07, 131.91  4.00, 7.90  -0.21, 3.78 | | -  25.66  3.47 | | -  <0.001***  >0.05 | | |
| Lay Date (n=784)   Mod 16 | *Intercept*  **Known years breeding as a pair (scaled, SD= 1.17)** | 135.10  -0.74 | | 133.53, 136.66  -1.27, -0.18 | | -  7.29 | | - 0.01* | | |  |
| Lay Date (n=784)     Mod 17 | *Intercept* **Average year (per breeding pair) (scaled, SD=4.67)**  **Deviation from average year (per breeding pair)**  **(scaled, SD=1.46)** | 135.02  -0.64  -0.78 | | 134.38, 135.62  -1.28, -0.02  -1.24, -0.36 | | - 4.00 12.61 | | - 0.015  <0.001*** | | |  |

**Table S3.** Results of a within-subject centring (van de Pol & Wright 2009) decomposition model used to distinguish between-individual and within-individual effects of laying date on breeding success. The model included each individual's mean laying date (between-individual effect) and annual deviations from that mean (within-individual effect). Shown are model coefficients, sample sizes, bootstrapped 95% confidence intervals, effect sizes, and *P*-values derived from likelihood ratio tests (χ²). Significant results are highlighted in bold; levels of statistical significance are indicated by asterisks (*P* < 0.05*, *P* < 0.01**, *P* < 0.001***).

| Response | Coefficient | Effect Size | Confidence Intervals | *χ^2^* | p value |
| --- | --- | --- | --- | --- | --- |
| Breeding success (n=1527, 733 individuals)      Mod 18 | Intercept **Average Lay Date (scaled, SD=6.45 days)**  Deviation from Lay Date (scaled, SD=4.61 days)  Year (scaled, SD=4.92y)  **Interactive term deviation and year (scaled)** | 1.12  -0.16  -0.07  0.06  0.16 | 0.98, 1.25  -0.28, -0.03  -0.18, 0.04  -0.07, 0.20  0.02, 0.29 | -  5.80   1.47   0.83  5.14 | - 0.015*   >0.05   >0.05  0.02* |

**Table S4.** Results of linear models testing whether annual mean laying date tracked interannual variation in phytoplankton bloom onset, and whether bloom onset and bloom amplitude changed over the study period in the Irish and Celtic Seas.

| Model | Model number | Estimate | Confidence intervals | Standard error | t value | p value |
| --- | --- | --- | --- | --- | --- | --- |
| Mean lay DOY~ Celtic Sea bloom onset DOY | Mod 19 | 0.14 | -0.02, 0.29 | 0.07 | 1.90 | 0.07 |
| Mean lay DOY~ Irish Sea bloom onset DOY | Mod 20 | 0.08 | 0, 0.15 | 0.03 | 2.16 | 0.049* |
| Celtic Sea amplitude~ year | Mod 21 | -0.04 | -0.07, -0.01 | 0.02 | -2.5 | 0.02* |
| Celtic Sea onset ~ year | Mod 22 | 0.28 | -0.90, 1.47 | 0.56 | 0.52 | >0.05 |
| Irish Sea amplitude ~ year | Mod 23 | -0.04 | -0.09,0.008 | 0.03 | -1.78 | >0.05 |
| Irish Sea onset ~ year | Mod 24 | -1.33 | -3.49, 0.82 | 1.01 | -1.32 | >0.05 |

**Table S5.** Supplementary analysis for the subset of individuals with laying date records in consecutive breeding seasons (n = 100), examining the effects of previous year's laying date on non-breeding duration and subsequent laying date. This analysis was conducted as post-breeding departure date and laying date are correlated but not equivalent measures, allowing assessment of whether observed migration–breeding relationships could reflect repeatability in breeding phenology. Shown are model coefficients, sample sizes, bootstrapped 95% confidence intervals, effect sizes, and *P*-values derived from likelihood ratio tests (χ²). Significant results are highlighted in bold; levels of statistical significance are indicated by asterisks (*P* < 0.05*, *P* < 0.01**, *P* < 0.001***).

| Response | Coefficient | | Effect Size | | Confidence Intervals | | *χ^2^* | | p value |
| --- | --- | --- | --- | --- | --- | --- | --- | --- | --- |
| Over-wintering duration  (n=100)   Mod 25 | *Intercept* Previous lay date (scaled, SD=6.26) | 209.04 -0.05 | | 204.68, 213.40 -2.77, 2.54 | | -  0.001 | | -  >0.05 | |
| Lay date  (n=100)     Mod 26 | *Intercept*  Mean over-wintering duration (scaled, SD=10.45)  Deviations from mean over-wintering duration (scaled, SD=8.99)  **Previous lay date (scaled, SD=6.26)** | 135.46  0.90  0.29  1.34 | | 131.94, 138.90  -0.32, 2.08  -0.39, 0.95  0.18, 2.51 | | -  2.13   0.98   3.42 | | - >0.05  >0.05   0.03* | |

**Table S6.** Supplementary analyses testing whether the relationships between first- and second-arrival dates and lay date differed according to the sex of the second-returning bird, and whether the arrival interval between pair members influenced lay date as a measure of within-pair arrival synchrony. Shown are model coefficients, sample sizes, bootstrapped 95% confidence intervals, effect sizes, and *P*-values derived from likelihood ratio tests (χ²). Significant results are highlighted in bold; levels of statistical significance are indicated by asterisks (P < 0.05*, P < 0.01**, P < 0.001***).

| Response | Coefficient | | Effect Size | | Confidence Intervals | | *χ^2^* | | p value |
| --- | --- | --- | --- | --- | --- | --- | --- | --- | --- |
| Lay date  (n=38; as 2 breeding pairs had both members of the pair arrive on the same day)   Mod 27 | *Intercept* **Late bird arrival date (DOY) (scaled, SD=12.89 )** Later sex (male)  Early bird arrival date (DOY)  (scaled, SD=12.60)  Late bird arrival date * sex (male)  Early bird arrival date * sex (male) | 137.28  5.25  -3.38  0.85  -4.40  2.66 | | 132.76, 141.73  1.13, 9.34  -7.44, 0.64  -3.09, 4.86  -12.46, 3.18  -4.37, 10.20 | | -  7.07  2.72  0.21  1.27  0.53 | | - 0.008**  >0.05 >0.05  >0.05  >0.05 | |
| Lay date  (n=40)     Mod 28 | *Intercept*  **Early bird arrival date (DOY)**  **(scaled, SD= 12.50)**  **Arrival interval (scaled, SD=7.09)** | 135.27 5.31   2.36 | | 131.29, 138.76  3.33, 7.14  0.58, 4.36 | | -  28.23  7.06 | | - <0.001***   0.008** | |

**Figure S1.** Sensitivity analysis of the effect of lay date on breeding success. Points show coefficient estimates (±95% CI) from mixed-effects models fitted to the full dataset (n=766) and to untracked birds only (n=376). Although the effects are no longer statistically significant when restricted to untracked birds, the direction and magnitude of the estimate are consistent, indicating reduced power rather than an effect of tracking devices.

**Figure S2.** Modelled relationships between breeding success and lay date plotted for each year of observation. Panels are shown to support mixed‐effects model outputs and to illustrate interannual variability in the relationship between breeding success and lay date.

**Figure S3.** Modelled relationships between chick peak mass and lay date plotted for each year of observation. Panels are shown to support mixed‐effects model outputs and to illustrate interannual variability in the relationship between breeding success and lay date.

**Figure S4.** Annual chlorophyll phenology metrics for the Celtic Sea. Smoothed (LOESS: span = 0.25) daily chlorophyll time series are shown for each year. Dashed vertical lines indicate the timing of the derivative-based bloom onset, and seasonal amplitude values are displayed in each panel. Metrics were calculated independently for each year.

**Figure S5.** Annual chlorophyll phenology metrics for the Irish Sea. Smoothed (LOESS: span = 0.25) daily chlorophyll time series are shown for each year. Dashed vertical lines indicate the timing of the derivative-based bloom onset, and seasonal amplitude values are displayed in each panel. Metrics were calculated independently for each year.

**Figure S6.** Spatial distribution of Manx shearwater foraging locations during incubation (blue) and chick-rearing (red) periods. Points represent a random subsample of GPS positions used to illustrate broad-scale foraging distributions. Shaded polygons indicate the Celtic Sea (green) and Irish Sea (blue) regions used for phytoplankton bloom analyses. Foraging locations occurred within both regions during both breeding stages, although the relative distribution of locations differed between incubation and chick-rearing.
